# A biostatistical approach for augmenting rare bianthraquinone antibiotic production by *Streptomyces* sp. RA-WS2 using Taguchi design

**DOI:** 10.1186/s13568-022-01497-5

**Published:** 2022-12-14

**Authors:** Ravi Singh Manhas, Amit Kumar, Asha Chaubey

**Affiliations:** 1 Fermentation & Microbial Biotechnology Division, CSIR-Indian Institute of Integrative Medicine, Canal Road, Jammu, 180001 India; 2 Quality Management & Instrumentation Division, CSIR-Indian Institute of Integrative Medicine, Canal Road, Jammu, 180001 India; 3 Academy of Scientific and Innovative Research, CSIR-Human Resource Development Centre, Campus Ghaziabad, Ghaziabad, 201002 India

**Keywords:** *Streptomyces*, Fermentation, 9,9’-bianthraquinone, Setomimycin, Optimization Taguchi design

## Abstract

Consistent production of bioactives from microbial sources remains a big challenge for fermentation based bio-processes. Setomimycin, a rare 9,9'-bianthrylanthracene antibiotic reported to be active against Gram positive bacteria i.e. *Staphyloccocus aureus*, *Bacillus subtilis*, *Bacillus cereus*, and *Mycobacterium smegmatis*, including mycobacteria is one of the least exploited antibiotic. Present work aims to enhance and maximize setomimycin production using One Factor at a Time (OFAT) approach, followed by Taguchi L9 orthogonal array (OA) design in 30L fermenter. Four most influential parameters, namely carbon source, nitrogen source, air and agitation were selected for optimization studies. The optimized production medium supplemented with 150 g/L glycerol and 7.5 g/L soyabean meal with an agitation rate of 100 RPM and air flow rate of 20 LPM (Liters Per Minute) resulted in 675 mg/L setomimycin production within 96–108 h of fermentation as compared to the initial production i.e. 40 mg/L. Thus, an overall enhancement of 16.8 folds was achieved in setomimycin production after optimization in 30L fermenter.

**Graphical Abstract:**

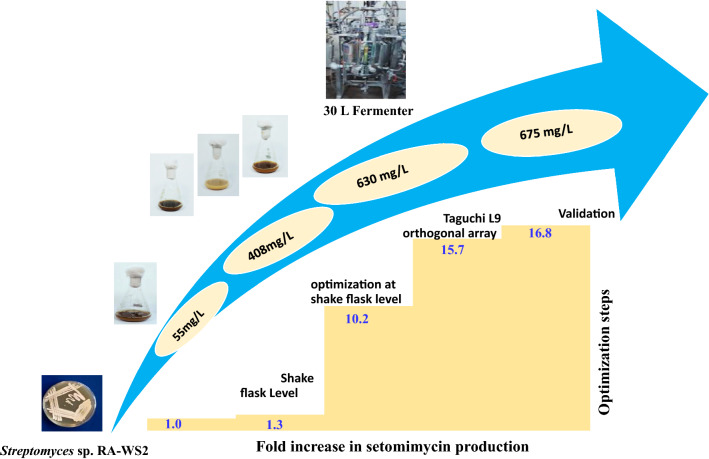

**Supplementary Information:**

The online version contains supplementary material available at 10.1186/s13568-022-01497-5.

## Introduction

**Scheme 1:**
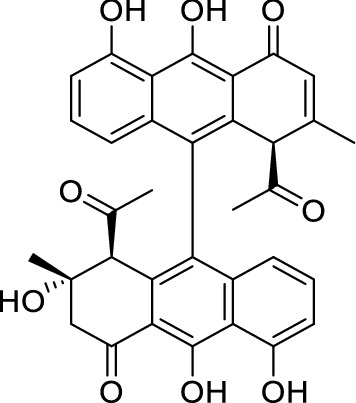
Structure of setomimycin

Increasing antibiotic resistance towards the existing drugs has surfaced as a major concern recently. Therefore, a continuous research and development for the discovery of new antibiotics has become a foremost goal for the scientists. Most of the antibiotics discovered so far are either the natural products of microbes or semi-synthesised from these products (Zhang et al., 2021). Biaryl preanthraquinones such as julichromes, spectomycins, and setomimycins, are special class of antibiotics isolated from actinobacteria specially, *Streptomyces* genus (Dong et al., 2020: Staley et al., 1994: Omura et al., 1978). Atroposelective biaryl compounds are mostly formed in nature through intermolecular oxidative phenol coupling (Wang et al., 2020). The monomeric subunits of these biarylic pre-anthraquinones are derived from a common polyketide precursor. The coupling of the two subunits takes place in regio-selective manner depending on the Streptomyces strain. Setomimycin, a rare 9,9'-bianthrylanthracene antibiotic (Scheme 1) was isolated for the first time from *Streptomyces pseudovenezuelae* by Omura et al. (1978). Although the total biosynthesis of setomimycin is not well understood, however Präg et al. (2014) identified the biosynthetic gene cluster for setomimycin biosynthesis in *S. afghaniensis* and suggested that cytochrome P450 is responsible for the dimerization. Setomimycin is reported to be active against various Gram-positive bacteria such as *B. subtilis, S. aureus, M. smegmatis* and *B. cereus.* Recently, our group has also reported a potential strain for setomimycin production and its bioactive potential as antiviral as well as anticancer agent (Manhas et al., 2022a, b).

Keeping in mind the limited market availability and cost, it is important to develop efficient fermentation process with enhanced and consistent production of setomimycin. Growth of microbes and production of their metabolites in a bioreactor is highly affected by physiochemical factors like nitrogen sources, carbon sources, trace elements. pH, temperature, time of fermentation, agitation and aeration rate also play significant role in the process (Wenzel et al., 2011). Raw material for fermentation medium involves the major cost of a process, therefore needs to be optimized strategically. The components of media should yield optimal growth of microbes along with higher production of the metabolite with no or minimum number of by-products (Elisashvili et al., 2001). Glycerol, being a major by-product of alcoholic beverage and soap industries is one of the obvious choice as a cheap carbon source. Soyabean meal is also a key agri-waste after the extraction of soy oil. Due to its low cost, high protein content and ease of availability, it is widely used as an alternative nitrogen source for fermentation (Hong et al., 2004; Song et al., 2008). Also, maintaining the required cultivation conditions keeping minimum stress exposure for the microorganism are the key factors in optimizing the fermentation process and further scale up (Schmidt, 2005). Optimization of these parameters during fermentation is an important step to surpass the difficulties arising due to large scale production of the desired metabolite.

Usual Design of Experiments (DOE) for developing a process generally requires a large set of experiments, which is highly tedious and time consuming. Therefore, in order to overcome this, a statistical approach by Taguchi design that consists of well-planned experiments has been suggested (Bhattacharya et al., 2009). Taguchi orthogonal array design allows the study of a set of variables with a limited number of experiments instead of large set of experiments. Thus, it helps in improving the quality and the performance of the process through DOE and Robust Technology Development (RTD). It utilises pair-wise balancing property, where variables are arranged in an orthogonal array and each combination of variables appears an equal number of times (Subba Rao et al., 2008). Various fermentation processes have been successfully optimized using Taguchi method (Azmi et al., 2011; Rao et al., 2004; Nagarijun et al., 2005). Analysis of Variance (ANOVA) is also used to validate the statistical significance of the experimental results, thus making the analysis more accurate and precise (Sawyer, 2009).

In the present study, we have used an actinobacterium *Streptomyces* sp. strain RA-WS2 that has recently been identified for setomimycin production (Manhas et al., 2022a, b). The culture parameters that could influence the production of setomimycin; such as carbon source, nitrogen source, agitation and aeration rate were optimized by methodological application of Taguchi design using an OA layout of L9. The results obtained were further validated by fermentation in 30L bioreactor to compare the predicted and observed values.

## Materials and methods

### Microorganism

Actinobacterium *Streptomyces* sp strain RA-WS2, isolated from soil sample collected from Shivalik Foothills 32.7266° N, 74.8570° E, India has been used in the present study. The culture has also been submitted to Microbial Type Culture Collection (MTCC- 25512) under Budapest Treaty. The isolate has recently been reported for the production of a bianthraquinone antibiotic i.e. setomimycin under submerged fermentation for its antiviral and anticancer properties by Manhas and coworkers (2022a, b).

### Preparation of inoculum and growth conditions

The actinobacterium strain *Streptomyces* sp. RA-WS2 was pre-cultured in Starch Casein Agar (SCA) medium (Additional file 1: Table S1a) and incubated at 28 ± 2 °C for 72 h. A loopful of freshly grown culture from the plate was inoculated in 500 mL Erlenmeyer flask with volume of 100 mL of pre-seed medium (Additional file 1: Table S1b) and incubated at 28 ± 2 °C for 48 h under shaking conditions to prepare pre-inoculum. Seed inoculum was prepared by transferring 10% of the pre-seed inoculum to 1000 mL flask with working volume of 300 mL (Additional file 1: Table S1c) and incubated at 28 ± 2 °C with 200 RPM agitation for 48 h. 10% seed inoculum was further transferred to production medium (Additional file 1: Table S1d) and allowed to grow at 28 ± 2 °C and 200 RPM for 96–144 h for production of setomimycin.

### Determination of biomass (Dry Cell Weight)

Fermented broth (50 mL) was centrifuged at 10,000×*g* for 10 min to separate the supernatant and cell pellet. The cell pellet was allowed to dry until constant weight was achieved. The results have been represented in terms of g/L.

### Extraction of bioactives

Post fermentation, the fermented broth was homogenized with 10% methanol for about 2 h and thereafter extracted at least thrice with equal volumes of ethyl acetate as described previously (Manhas et al., 2022b). The organic phase thus obtained was concentrated on a rotary evaporator at 50 °C to get crude extract. Extraction yields for crude extract were calculated in terms of g/L.

### HPLC profile and quantification of Setomimycin

HPLC profiling of crude extract was carried out using HPLC system (Shimadzu, UFLC) consisting of a Quaternary pump with vacuum degaser, thermostat column compartment, autosampler, and PDA detector. Reverse-phase column (Merck RP; 18e Lichrosphere, 250*4 mm, 5 µm) was used for profiling and quantification of setomimycin (Additional file 1: Figure S1(a)). Mobile phase consisted of Solution A: (Water with 0.1% Formic acid) Solution B: (Acetonitrile) and the column temperature was kept at 40 °C during analysis and quantification was carried out at 265 nm with gradient elution during analysis (Additional file 1: Table S2).

The samples for quantification were prepared by dissolving crude extracts in methanol at concentration of 1 mg/ mL. The standard curve was prepared with different dilutions of stock of setomimycin (≥ 97% purity) and quantification was done using standard curve (Additional file 1: Figure S1(b). The solution was filtered through 0.45 mμ membrane filter and degassed in a sonicator for 3 min. The injection volume used was 5µL and flow rate was kept at 0.75 mL/min.

### Optimization studies using One Factor at a Time (OFAT) approach

Present study was designed to observe responses in terms of biomass (DCW) and setomimycin content. Various physical and chemical factors were evaluated on the basis of the two responses such as biomass (DCW g/L) and setomimycin (mg/L) production. Initially, the effect of various production media (Additional file 1: Table S1d) was studied to determine growth and production of setomimycin. Further studies on the effect of temperature (20 to 40 °C), pH (5 to 9), inoculum (2.5 to 15%) in selected fermentation medium with agitation (50 to 300 rpm) and different air to volume ratio were carried out. Effect of various carbon and nitrogen sources was evaluated under the optimized conditions. Optimization studies were carried out in 500 mL Erlenmeyer flasks containing 100 mL production medium and incubated at 28 ± 2 °C for 96–120 h on a rotary shaker at 200 RPM. All the optimization experiments were carried out in triplicate and responses in terms of biomass (DCW, g/L), crude extract (g/L) and setomimycin content (mg/L) were determined.

### Taguchi orthogonal array design layout in 30 L fermenter

Use of Taguchi approach in Design of Experiments (DOE) facilitates the entire parameter space with a small number of experiments. Therefore, L9 Taguchi orthogonal array was chosen to design the experimental layout. Based on the significant factors identified in OFAT experiments, carbon source (glycerol), nitrogen source (soyabean meal), agitation (RPM) and air (VVM) were selected for optimization studies at fermenter level (30 L fermenter). Taguchi orthogonal array experimental design led to 9 experiments in 30 L fermenter having 15 L production medium. For the fermentation experiments in the bioreactor, three levels were attributed for each independent parameter. The experimental design based on the significant parameters and levels as shown in Table 1.

**Table 1:** Assignment of factors and levels of the orthogonal array design L9 for optimization of biomass (DCW) and setomimycin production by *Streptomyces* sp. RA-WS2

Factors	Level-1	Level-2	Level-3
Carbon source (Glycerol g/L)	50	100	150
Nitrogen source (Soyabean meal g/L)	2.5	5.0	7.5
Air (LPM)	10	15	20
Agitation (rpm)	100	150	200

The experimental matrix (L9) with four significant independent factors and their respective levels have been illustrated in Additional file 1: Table S3. For each experiment, the responses in terms of biomass (DCW g/L) and setomimycin content (mg/L) were evaluated. The mean responses for each run in the array were analyzed in terms of Signal- to- noise (S = N) ratio to study the variation. Analysis of the graphs and ANOVA was carried out using Minitab (version 20) in terms of S/N ratio as larger the best using following equation 

$${{{S}} \mathord{\left/ {\vphantom {{\text{S}} {\text{N}}}} \right. \kern-\nulldelimiterspace} {{N}}} = \, - {\text{10 *log}} \Big(\sum(1/Y^2)/n\Big)$$

where, S/N = Signal to noise ratio. Y = Signal factor (Biomass DCW in g/L or Setomimycin content in mg/L). n =  Number of repetitions in the experiment.

Taguchi design recommends the use of the S/N ratio to determine which settings of the controllable factors result in a closer mean to the desired target and a minimum variability transmitted from the noise variables (Yang and Tarng, 1998; Freddi and Salmon, 2019). The significance of all terms was judged statistically when the *p* value was less than 0.05 (*p* < 0.05).

## Results

Pre-requisite for the optimization of a fermentation process is to choose suitable and cost-effective growth medium. Further optimization of physico-chemical parameters on DCW and setomimycin production by *Streptomyces* sp. RA-WS2 were carried out in the selected medium after using OFAT experiments.

### Effect of production media

**Fig. 1:**
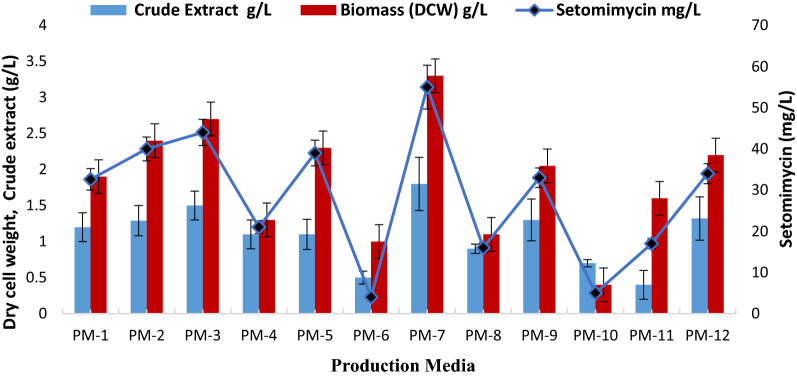
Effect of production media on fermentation of *Streptomyces* sp. RA-WS2

In order to evaluate the effect of growth media, *Streptomyces* sp. strain RA-WS2 was grown in various media and DCW as well as setomimycin content were evaluated. As shown in Fig. 1, PM-7 (Constituents in g/L: Glycerol 100.0; soyabean meal 5.0; CaCO_3_ 3.0; K_2_HPO_4_ 1.0; MgSO_4_ 1.0; NaCl 2.0 pH − 7.0 ± 0.2) was observed to be the best medium for the growth of the culture as well as setomimycin production. Therefore, it was chosen as the preferred fermentation production medium for further optimization experiments.

### Effect of fermentation conditions

**Fig. 2:**
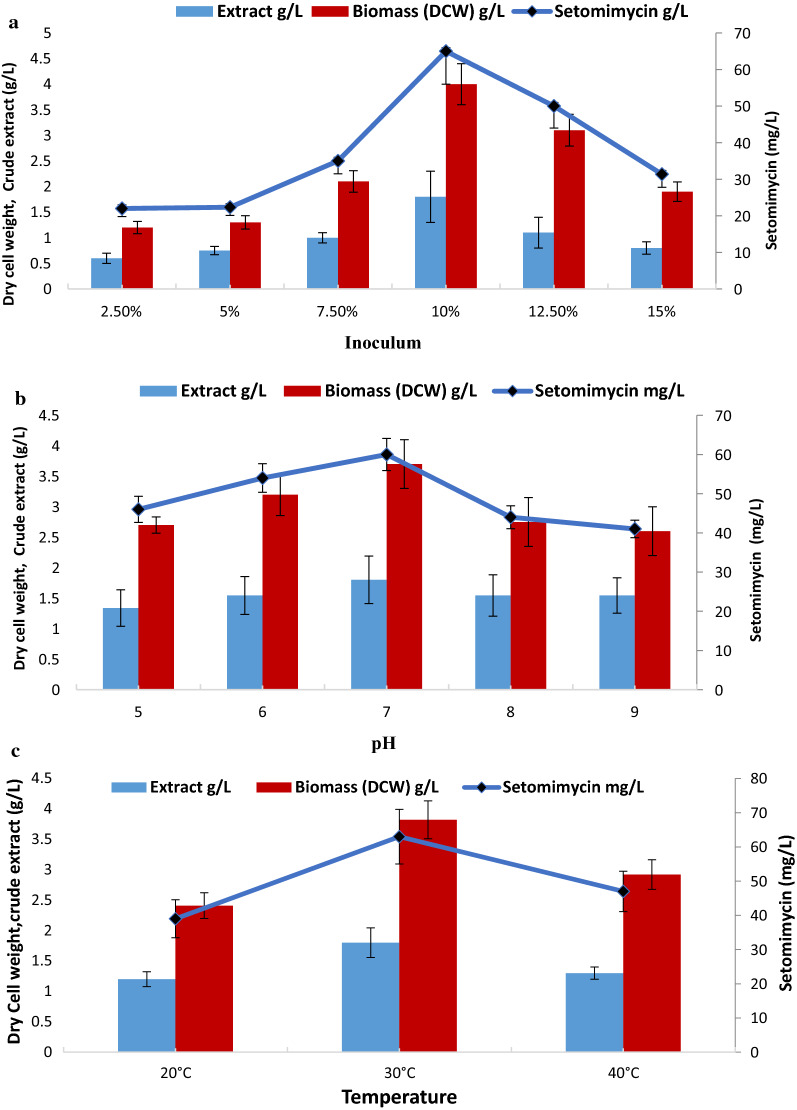

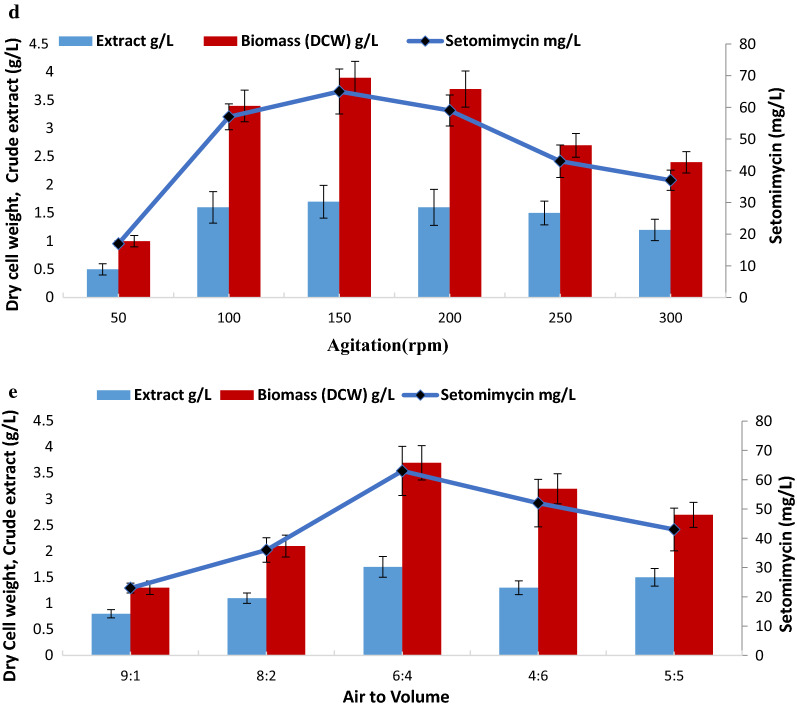
Effect of fermentation conditions on setomimycin production by *Streptomyces* sp. RA-WS2 (**a**) Inoculum percentage (**b**) pH (**c**) Temperature (**d**) Agitation (**e**) Air to volume ratio

Figure 2 represents the effect of fermentation conditions on DCW as well as setomimycin production. As shown in Fig. 2a, 10% inoculum was found to be the best for optimum DCW (4.0 g/L) as well as setomimycin production (65 mg/L). The effect of pH on DCW and production of setomimycin, was studied by growing culture over a broad pH range i.e. pH 5.0 to 9.0. It has been seen from Fig. 2(b), that highest biomass (3.7 g/L) was obtained at pH 7.0, while setomimycin production was highest at pH 6.5–7.5 (60 mg/L), although production was observed at wide pH range (pH 5.0 to 9.0). Likewise, fermentation of *Streptomyces* sp. RA-WS2 at various temperature range from 10 °C to about 45 °C revealed that the optimum production (DCW as well as setomimycin production) was achieved at 30 °C (Fig. 2c). To study the effect of agitation, the *Streptomyces* sp. RA-WS2 was grown on selected production medium with agitation range from 50 to 400 rpm and it was observed that agitation from 100 to 300 rpm supports the fermentation with maximum production of setomimycin with 10% seed inoculum (Fig. 2d). Fermentation in shake flask experiments also revealed that moderate air is required by *Streptomyces* sp. RA-WS2 for growth and production of setomimycin (Fig. 2e).

### Effect of carbon source

**Fig. 3:**
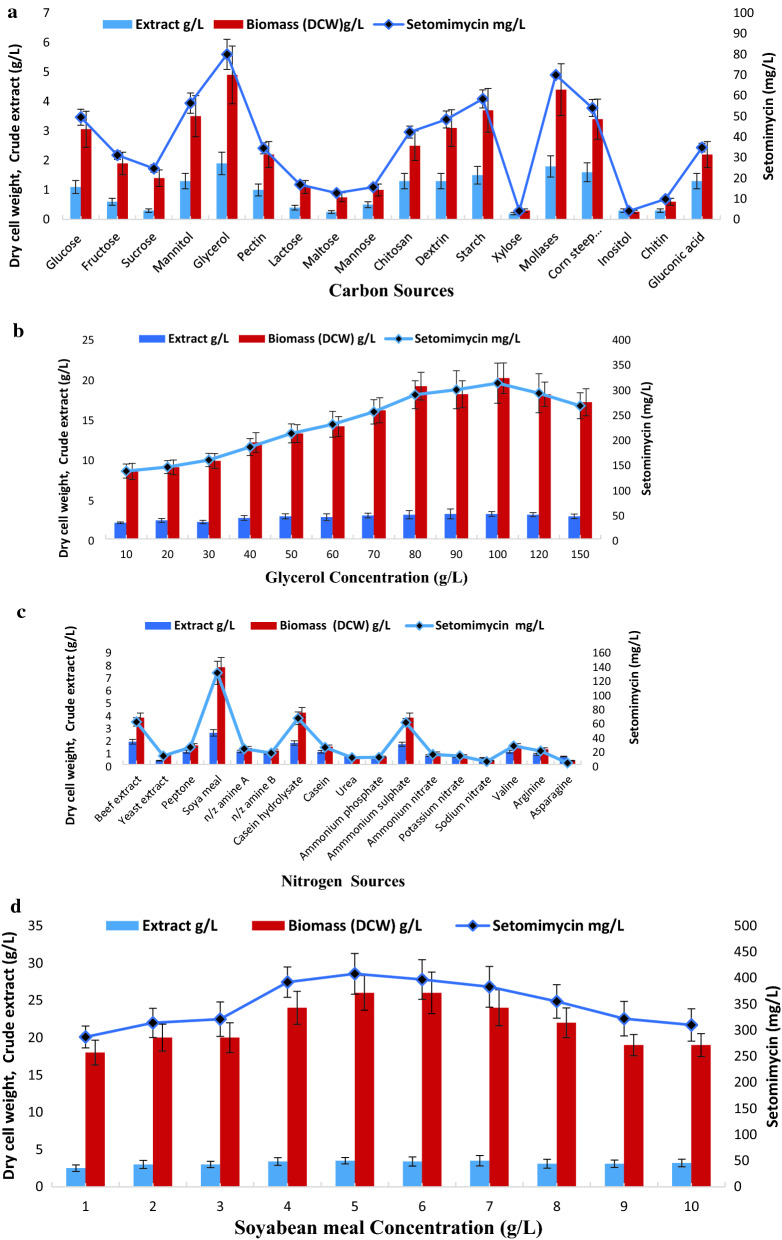
Effect of carbon and nitrogen sources on setomimycin production by *Streptomyces* sp. RA-WS2 (**a**) Effect of various carbon sources (**b**) Effect of glycerol concentration as carbon source (**c**) Effect of various nitrogen sources (**d**) Effect of soyabean meal concentration as nitrogen source

In order to evaluate the effect of carbon sources, several monosaccharides, disaccharides and polysaccharides were supplemented as carbon source in the selected production medium i.e. PM-7 (Constituents in g/L Starch-10.0; CaCO_3_ −3.0; K_2_HPO_4_ −1.0; (NH_4_)_2_ SO_4_; −2.0 MgSO_4_−1.0; NaCl-1.0, pH 7 ± 0.2). Among the used carbon sources as shown in Fig. 3a, it was observed that glycerol served as the best carbon source followed by molasses and corn steep liquor. Concentrations of glycerol in production medium was optimized by using varied concentrations used during fermentation experiments to evaluate DCW and setomimycin concentration. Figure 3b demonstrates that DCW as well as setomimycin concentration increased with increasing concentration of glycerol with an optimum production with 100 g/L glycerol supplementation in the production medium. Thereafter, no significant increase in DCW or setomimycin production was observed.

### Effect of nitrogen source

Based on the previous experiments, 100 g/L glycerol was supplemented as carbon source to optimize the nitrogen source in the production medium. Various organic and inorganic nitrogen sources were screened for growth and setomimycin production as shown in Fig. 3c. Results demonstrate that soyabean meal followed by beef extract and casein hydrolysate result in maximum DCW (7 g/L) as well as setomimycin production (129 mg/L). Further, to check the effect of concentration of nitrogen source (Soyabean meal) on production of setomimycin varied concentrations of soyabean meal (1 to 10 g/L) were used for fermentation. It was observed (Fig. 3d) that 5–6 g/L was the best for achieving the highest DCW (26 g/L) as well as setomimycin (408 mg/L) production. Further optimization of fermentation conditions was carried out in 30L Fermenter using modified PM-7 medium (Constituents in g/L: Glycerol 100.0; soyabean meal 5.0; CaCO_3_ 3.0; K_2_HPO_4_ 1.0; MgSO_4_ 1.0; NaCl 2.0, pH 7 ± 0.2).

### Taguchi orthogonal array design for optimization of biomass and setomimycin production in 30L fermenter

Based on the shake flask experiments using OFAT approach, significant factors were identified that had promising impact on fermentation of *Streptomyces* sp. strain RA-WS2. Elucidation of the experimental data revealed that four significant factors viz. glycerol, soyabean meal, agitation and air played a significant role on the growth (DCW) as well as setomimycin content. Three level, four factor Taguchi L9 orthogonal matrix was used keeping the optimum conditions of each factor as middle level (Table 1) and response was evaluated in terms of DCW (g/L) and setomimycin content (mg/L) with respect to time in each experiment. Table 2 demonstrates the results of the responses for 9 experiments in 30L fermenter. The DCW for various experiments varied from 26 g/L to 38 g/L, while setomimycin production was observed to be in the range from 420 mg/L to 630 mg/L. Optimum DCW as well as setomimycin content (630 mg/L) were observed in Exp 9 in 108 h.

**Table 2:** Response table of the orthogonal array design L9 for biomass (DCW) and setomimycin production by *Streptomyces* sp. RA-WS2

Run	Factor 1 Glycerol (g/L)	Factor 2 Soyabean meal (g/L)	Factor 3 Air (LPM)	Factor 4 Agitation (RPM)	Setomimycin (mg/L)	Biomass (DCW g/L)
1	50	2.5	10	100	420	26
2	50	5	15	150	465	29
3	50	7.5	20	200	500	31
4	100	2.5	15	200	390	24
5	100	5	20	100	615	37
6	100	7.5	10	150	610	35
7	150	2.5	20	150	500	31
8	150	5	10	200	515	32
9	150	7.5	15	100	630	38

### Taguchi analysis for DCW production

**Fig. 4:**
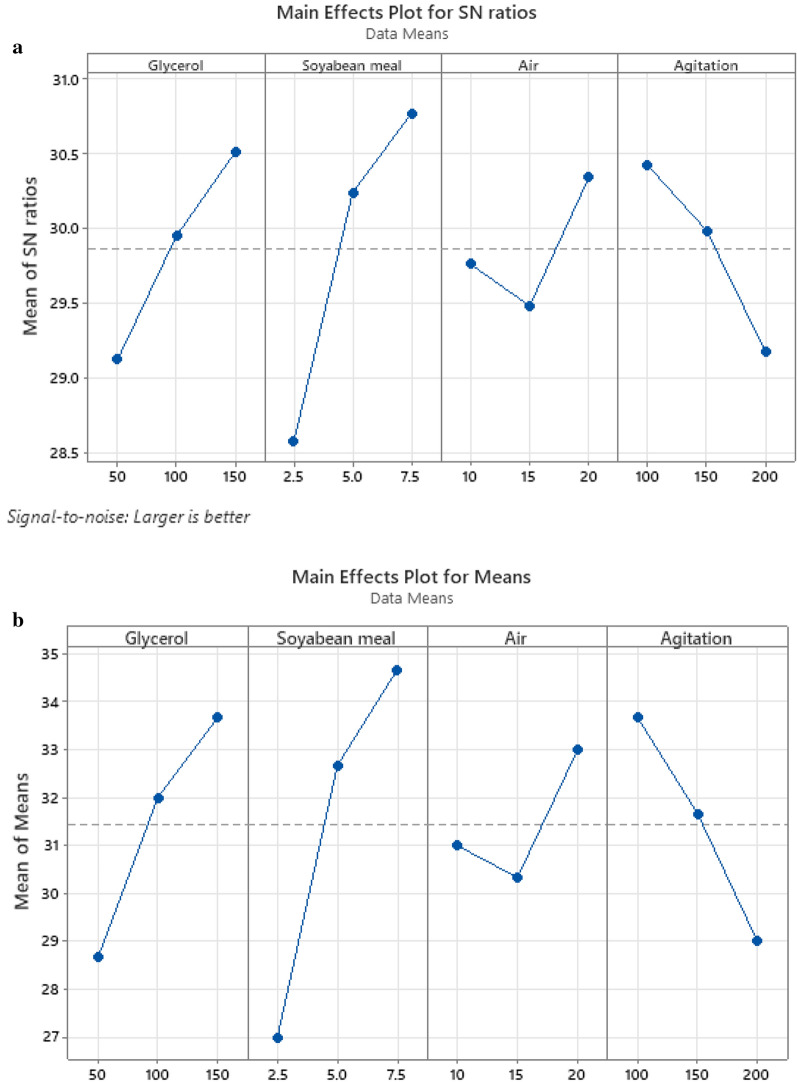

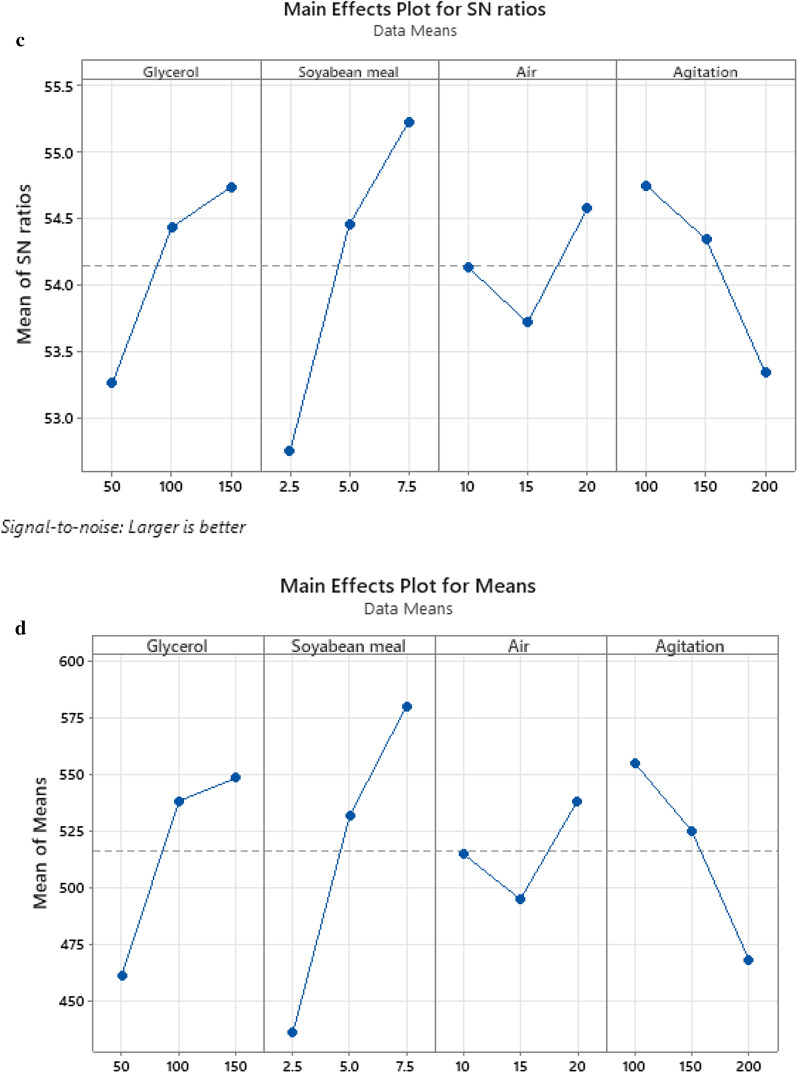
Main effects plot for Biomass (DCW) and Setomimycin production by *Streptomyces* sp. RA-WS2 (**a**) S/N ratio for Biomass (DCW) (**b**) Means for Biomass (DCW) (**c**) S/N ratio for Setomimycin (**d**) Means for Setomimycin content

S/N ratio analysis for DCW as shown in Table 3, revealed that the optimum condition for biomass production was similar to that obtained using main effects plot in Table 4 (150 rpm, 15 LPM, 150 g/L glycerol, and 7.5 g/L soyabean meal) of the fermentation variable. It was observed that soyabean meal was the highest influencing parameter in improving the production of biomass (Fig. 4a, b). The factor soyabean meal concentration with greatest delta (∇) value of 2.19 was assigned rank 1 depicting its highest effect on setomimycin production. Similarly, concentrations of glycerol and agitation rate rank second and third respectively. The aeration rate had the least effect on biomass (DCW) production and was assigned rank 4.

**Table 3:** Response Table for Signal to Noise Ratios of biomass (DCW: g/L) production by *Streptomyces* sp. RA-WS2

Level	Glycerol	Soyabean meal	Air	Agitation
1	29.12	28.58	29.76	30.42
2	29.95	30.24	29.48	29.99
3	30.51	30.77	30.34	29.18
Delta (∇)	1.38	2.19	0.86	1.24
Rank	2	1	4	3

**Table 4:** Response Table for Means of biomass (DCW: g/L) production by *Streptomyces* sp. RA-WS2

Level	Glycerol	Soyabean meal	Air	Agitation
1	28.67	27.00	31.00	33.67
2	32.00	32.67	30.33	31.67
3	33.67	34.67	33.00	29.00
Delta (∇)	5.00	7.67	2.67	4.67
Rank	2	1	4	3

### Taguchi analysis for Setomimycin production

S/N ratio analysis revealed that the optimum condition for setomimycin production was similar to that obtained using main effects plot of the fermentation. It is evident from the S/N and the main effects plots (Larger the better) that the concentration of soyabean meal had the greatest effect on setomimycin production followed by glycerol concentration, agitation, and aeration rate (Fig. 4c, d). As shown in Tables 5 and 6, the factor soyabean meal concentration having greatest delta (∇) value i.e., 2.47 was assigned rank 1 depicting its highest effect on setomimycin. Similarly, concentrations of glycerol and agitation rate were assigned rank second and third respectively. The aeration rate had the least effect on setomimycin production and was therefore assigned rank 4.

**Table 5:** Response Table for Signal to Noise Ratio of Setomimycin production by *Streptomyces* sp. RA-WS2

Larger is better				
Level	Glycerol	Soyabean meal	Air	Agitation
1	53.26	52.76	54.14	54.74
2	54.44	54.45	53.72	54.35
3	54.73	55.22	54.58	53.35
Delta (∇)	1.47	2.47	0.86	1.40
Rank	2	1	4	3

**Table 6:** Response Table for Means of Setomimycin production by *Streptomyces* sp. RA-WS2

Level	Glycerol	Soyabean meal	Air	Agitation
1	461.7	436.7	515.0	555.0
2	538.3	531.7	495.0	525.0
3	548.3	580	538.3	468.3
Delta (∇)	86.7	140.3	43.3	86.7
Rank	2.5	1	4	2.5

### Regression equation and analysis of Variance (ANOVA)

Linear regression model was used to fit the experimental data and to identify the relevant model terms. The regression equations for DCW and setomimycin production have been shown below:

DCW (g/L) = 22.78 + 0.0500 Glycerol + 1.533 Soyabean meal + 0.200 Air—0.0467 Agitation.

Setomimycin (mg/L) = 381.1 + 0.867 Glycerol + 28.67 Soyabean meal + 2.33 Air—0.867 Agitation.

ANOVA was used to further analyse the results of orthogonal array experiments and to find out the variation of each of the factor towards the growth (DCW) and production of setomimycin. The factors i.e., soyabean meal, glycerol and agitation bearing p < 0.05 were identified as significant ones. Lowest P-value and highest F-value in case of soyabean meal also shows its highest influence over the production of cell biomass as shown in Table 7. Similarly, the ANOVA (Table 8) demonstrates the contribution of each fermentation variable depicting that the production of setomimycin is significantly influenced by concentration of soyabean meal followed by concentration of glycerol, agitation rate and aeration rate respectively. Lowest P-value and highest F-value in case of soyabean meal also shows its highest influence over the production of setomimycin.

**Table 7:** Analysis of Variance for Biomass (DCW) production by *Streptomyces* sp. RA-WS2

Source	DF	Adj SS	Adj MS	F-Value	P-Value
Regression	4	164.333	41.083	11.83	0.017
Glycerol	1	37.500	37.500	10.80	0.030
Soyabean meal	1	88.167	88.167	25.39	0.007
Air	1	6.000	6.000	1.73	0.259
Agitation	1	32.667	32.667	9.41	0.037
Error	4	13.889	3.472		
Total	8	178.222

**Table 8:** Analysis of Variance for setomimycin production by *Streptomyces* sp. RA-WS2

Source	DF	Adj SS	Adj MS	F-Value	P-Value
Regression	4	54,166.7	13,541.7	9.55	0.025
Glycerol	1	11,266.7	11,266.7	7.95	0.048
Soyabean meal	1	30,816.7	30,816.7	21.73	0.010
Air	1	816.7	816.7	0.58	0.490
Agitation	1	11,266.7	11,266.7	7.95	0.048
Error	4	5672.2	1418.1		
Total	8	59,838.9			

### Interactions of variables and contour plots

**Fig.5:**
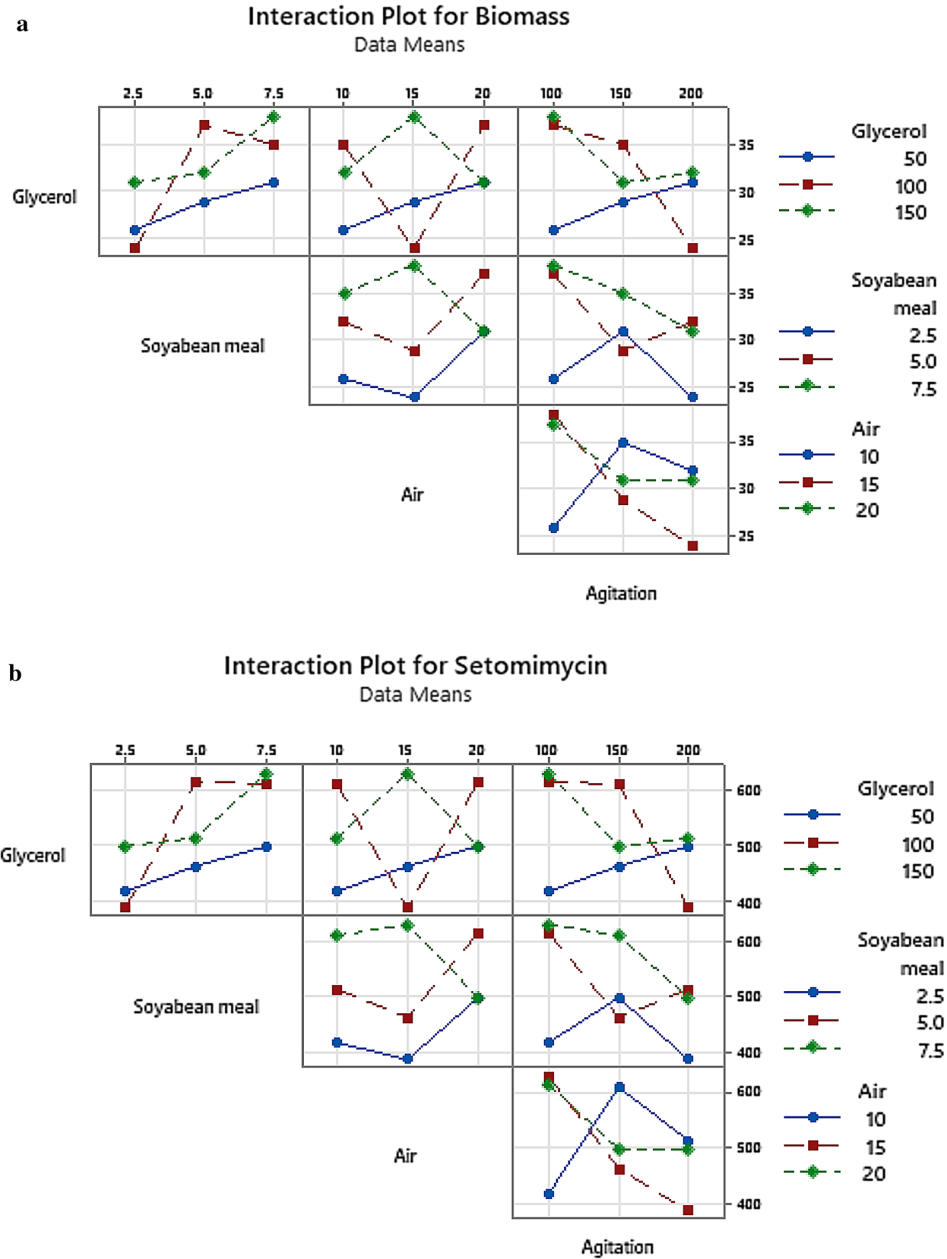

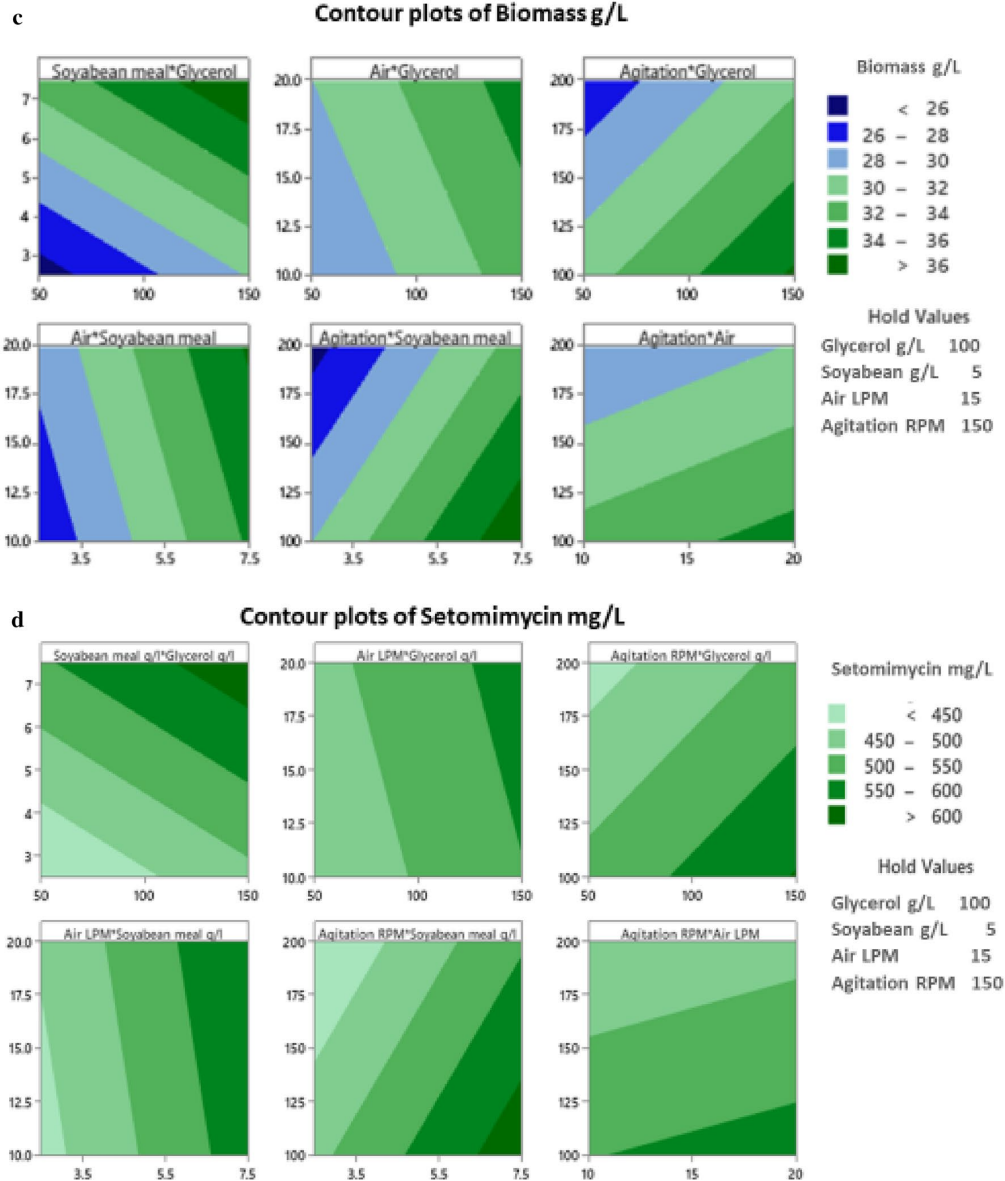
Interaction plots of various factors on (**a**) Biomass (DCW) (**b**) setomimycin production by *Streptomyces* sp. RA-WS2 and Contour plots for (**c**) Biomass (DCW) (**d**) Setomimycin production by *Streptomyces* sp. RA-WS2

The interaction between every two factors was evaluated as shown in Interaction plots for DCW (Fig. 5a) and setomimycin production (Fig. 5b), wherein X axes represent the level of first factor and lines are shown for the levels of other factor. S/N ratios are shown in the Y axes that demonstrate the interaction between the two factors by non-parallel lines. Figure 5c shows the contour plots explaining the relation between the process parameters and biomass production. It can be observed that high concentrations of glycerol as well as soyabean meal leads to high cell biomass. Similarly, high biomass production could be attained at high aeration rate and high glycerol concentration, while low agitation rate and high glycerol concentration gives high biomass. It can also be seen that higher aeration rate and high concentration of soyabean meal leads to high biomass production, whereas high production is also achieved with low agitation rate and high concentration of soyabean meal. Figure 5d demonstrates the contour plots explaining the relation between the process parameters and setomimycin production. It was found that higher concentrations of glycerol as well as soyabean meal leads to high production of setomimycin. Similarly, high production could be attained with higher aeration rate and high glycerol concentration, while lower agitation with high glycerol concentration support higher yield of setomimycin. It was also observed that higher aeration rate and high concentration of soyabean meal leads to high production of setomimycin, whereas high setomimycin production with low agitation rate and high concentration of soyabean meal. On the other hand, high aeration rate with low agitation supports more production of setomimycin.

### Prediction and validation of optimum fermentation conditions

After determining the optimal parameters, Taguchi model suggested solution for optimum production of DCW as well as setomimycin production followed by validation experiments. Table 9 represents the experimental values as compared to those predicted by the model. It was predicted that 150 g/L glycerol with 7.5 g/L soyabean meal with 10 LPM air at 100 rpm in 30L fermenter may results in 41 g/L DCW and 686 mg/L setomimycin. Fermentation experiments were carried out in 30L fermenter having 15L production medium by supplementing 10% innoculum and sampling was done after every 12 h. Additional file 1: Figure S2 demonstrates that maximum DCW i.e. 40 g/L and 675 mg/L setomimycin was obtained in 120 h. The experimental values were found to be close to the predicted values. A summary of optimization studies with fold increase in DCW and setomimycin has been compiled in Table 10.

**Table 9:** Validation of Taguchi experimental data values for biomass (DCW) and setomimycin production by *Streptomyces* sp. RA-WS2

Solution	Glycerol (g/L)	Soyabean meal (g/L)	Air (LPM)	Agitation (rpm)	Predicted Values		Experimental value	
					Biomass (DCW g/L)	Setomimycin (mg/L)	Biomass (DCW g/L)	Setomimycin (mg/L)
1	150	7.5	10	100	41	686	40 ± 5.1	675 ± 48

**Table 10:** Summary of optimization studies for biomass (DCW) and setomimycin production by *Streptomyces* sp. RA-WS2

Optimization stage	Medium	Biomass (DCW g/L)	Fold increase in Biomass (DCW) production	Setomimycin (mg/L)	Fold increase in setomimycin production
Initial fermentation conditions	PM-2	2.4	1	40	1
Medium optimization	PM-7	3.3	1.3	55	1.3
Optimization of physical parameters	PM-7	3.7	1.54	63	1.5
Optimization of carbon and nitrogen source	Modified PM-7	26	11	408	10.2
Optmization by Taguchi L9 othogonal array design	Modified PM-7	40	16.6	675	16.8

## Discussion

Biaryl preanthraquinones represented by julichromes, spectomycins, and setomimycin, are classified in literature on the basis of position of intermolecular oxidative phenol coupling of their monomeric units (Zhang et al., 2021). The natural process for biosynthesis of these compounds takes place from a common polyketide precursor, however the coupling reaction proceeds in a regioselective manner, with the position of attachment of the two subunits depending on the specific Streptomycete strain (Präg et al., 2014). Setomimycin is known to have antimicrobial activity against various Gram-positive bacteria such as *B. subtilis, S. aureus, M. smegmatis* and *B. cereus* (Omura et al., 1978). Although few groups have reported the isolation and bioactivities of setomimycin from different *Streptomyces* strains (Präg et al., 2014; Omura et al., 1978), however could not be commercially exploited much due to its limited access in the market. Recently, our group has reported a potential *Streptomyces* strain for the production of setomimycin and its bioactivities (Manhas et al., 2022a, b). Keeping in view the lower yields of production by the reported strains (Kay et al., 1981), attempts were made to develop an efficient fermentation process for the production of setomimycin.

Taguchi orthogonal array design facilitates with a set number of trials with only limited experiments (Bhattacharya et al., 2009). Therefore, an improved fermentation process may be developed by effective and robust Taguchi Experiment design. During the present optimization studies for the production of setomimycin by *Streptomyces* sp. RA-WS2 were performed starting from initial shake flask experiments with 2.6 g/L DCW with 40 mg/L setomimycin production. Further optimization strategy was designed to enhance setomimycin production with organized experimentation. Pre-requisite for the optimization of a fermentation process is to choose suitable and cost-effective growth medium (Singh et al., 2017). Several researchers prefer to use industrial byproducts so as to make the process commercially viable (Sitepu et al., 2014). Therefore, in order to maximize the DCW as well as setomimycin production, effect of production media was evaluated. Our results indicate that PM-7 (Starch casein broth) was the most favorable medium for growth as well as setomimycin production with 1.3-fold enhancement.

Further optimization of fermentation conditions in the chosen production medium, resulted in 3.7 g/L DCW and 63 mg/L setomimycin production with an overall 1.5 fold enhancement. Following optimization of fermentation conditions, carbon and nitrogen sources were optimized utilizing cheap and readily available industrial wastes. Interestingly, the selected carbon source i.e. glycerol, a readily available industrial byproduct as well as agriwaste nitrogen source, served the purpose of minimizing the cost of raw material (Yazdani et al., 2007). The optimization of carbon and nitrogen sources in the production medium led to 11 folds increase in DCW and 10.2 folds enhancement in setomimycin production with 100 g/L glycerol and 5 g/L soyabean meal in the modified production medium.

Based on the shake flask experiments using OFAT approach, four significant factors viz. glycerol, soyabean meal, agitation and air played a significant role on the growth (DCW) as well as setomimycin content. Taguchi method helps to attain the most optimal process variables in an easy, structured and an effective way (Shahavi et al., 2016). Further optimization of fermentation conditions was carried out in 30L Fermenter using modified PM-7 medium. The results indicate that the maximum setomimycin production (630 mg/L) is achieved in early stationary phase i.e. 108 h of fermemtation. Thus, Taguchi Orthogonal array optimization resulted in about 15.8 folds increase in DCW and 15.7 folds enhanced production of setomimycin. The results were in agreement with the reports demonstrating a multi fold increase in the bioproducts formation by optimization studies using Taguchi orthogonal array (Azmi et al., 2011; Rao et al., 2004; Nagarijun et al., 2005).

Our results also show that soyabean meal was the highest influencing parameter in improving the production of biomass (Fig. 4a,b). It is evident from the S/N and the main effects plots that the concentration of soyabean meal had the greatest effect on biomass production followed by glycerol concentration, agitation, and aeration rate. S/N ratio analysis revealed that the optimum conditions for setomimycin production were similar to that obtained using main effects plot of the fermentation. It is also evident from the S/N and the main effects plots (Larger the better) that the concentration of soyabean meal had the greatest effect on setomimycin production followed by glycerol concentration, agitation, and aeration rate. A successful fermentation process is influenced by various variables, therefore, effect of each factor on the production of a metabolite has to be understood deeply. Linear regression model was used to fit the experimental data and to identify the relevant model terms. Analysis of Variance (ANOVA) is also used to validate the statistical significance of the experimental results, thus making the analysis more accurate and precise (Sawyer, 2009). Therefore, ANOVA was used to further analyse the results of orthogonal array experiments and to find out the variation of each of the factor towards the growth (DCW) and production of setomimycin. In order to determine the quality of experimental results, statistical F-ratio was utilized, which is measured as the ratio of mean sum of squares (Kwak, 2005). Signal to noise ratio was calculated, where systemic variance was divided by unsystematic one and the larger ratio was considered better. Soyabean meal, glycerol and agitation bearing p < 0.05 were identified as significant factors for the optimization studies. Further, the optimization of fermentation process to maximize biomass (DCW) as well as setomimycin production not only required to study the effect of individual factors but also the interactions between these factors. Therefore, the interaction between every two factors was evaluated as shown in Interaction plots for DCW and setomimycin production (Fig. 5a). Contour plots examine the relation between the response variable and two control variables by viewing discrete contours of the predicted response variables. The contour plots explaining the relation between the process parameters and biomass as well as setomimycin production. Such relationships were found to have similar trend with that of reported for other antibiotics (Wang et al., 2008; El-Housseiny et al., 2021). After determining the optimal parameters, Taguchi model suggested solution for optimum production of DCW as well as setomimycin production followed by validation experiments. Therefore, fermentation experiments were carried out in 30L under suggested conditions and 40 g/L of DCW and 675 mg/L of setomimycin was obtained in 120 h of fermentation. This is the best yields for setomimycin production as compared to other *Streptomyces* sp. reported in literature (Kay et al., 1981). The experimental values were not only close to the predicted values, but also provided an optimized process parameters resulting in an overall 16.8 folds enhancement in the DCW and 16.6 folds enhancement in setomimycin production as compared to the unoptimized parameters. To the best of our knowledge, there are no reports on such study for setomimycin production by any *Streptomyces* culture.

In conclusion, process parameters for fermentation of *Streptomyces* sp. RA-WS2 have been optimized to maximize the biomass (DCW) and setomimycin production (mg/L). Taguchi orthogonal array design resulted an optimum yields of 40 g/L DCW and 675 mg/L setomimycin production in modified production medium. An overall 16.6 folds enhancement in DCW and 16.8 folds enhancement in setomimycin production was achieved after optimization. To our knowledge the present process for setomimycin production is the best production obtained so far in literature. It is anticipated that the demonstrated process may further be scaled up at commercial scale leading to increased availability of the antibiotic in the market.

## Supplementary Information

**Additional file 1: Table** **S1a**. Medium composition for maintenance of *Streptomyces* sp. RA-WS2. **Table S1b**. Medium composition for pre-inoculum preparation. **Table** **S1c**. Medium composition for inoculum preparation. **Table** **S1d**. Components of various media used for fermentation of Streptomyces sp. RA-WS2 for setomimycin production. **Table** **S2**. Gradient elution of mobile phase for quantification of setomimycin by HPLC. **Table** **S3**. Assignment of experimental conditions in the orthogonal array design L9 for culture in 30L fermenter. **Figure** **S1(a**). HPLC chromatogram of crude extract of Streptomyces sp. RA-WS2. **Figure** **S1(b)**. Standard curve of setomimycin (purity >97%) used for quantification of setomimycin in the crude extract of Streptomyces sp. RA-WS2. **Figure** **S2**. Time course of fermentation of Streptomyces sp. RA-WS2 and setomimycin production for validation of optimized conditions

## Data Availability

The research data generated and/or analyzed during the current study are available upon reasonable request from the corresponding author.
