# Supplementary material for: A biostatistical approach for augmenting rare bianthraquinone antibiotic production by Streptomyces sp. RA-WS2 using Taguchi design
Source: AMB Express. 2022 Dec 14;12:155. doi: 10.1186/s13568-022-01497-5 (PMC9751253; doi:10.1186/s13568-022-01497-5)
Supplement: Supplementary file 1 — Additional file 1: Table S1a. Medium composition for maintenance of Streptomyces sp. RA-WS2. Table S1b. Medium composition for pre-inoculum preparation. Table S1c. Medium composition for inoculum preparation. Table S1d. Components of various media used for fermentation of Streptomyces sp. RA-WS2 for setomimycin production. Table S2. Gradient elution of mobile phase for quantification of setomimycin by HPLC. Table S3. Assignment of experimental conditions in the orthogonal array design L9 for culture in 30L fermenter. Figure S1(a). HPLC chromatogram of crude extract of Streptomyces sp. RA-WS2. Figure S1(b). Standard curve of setomimycin (purity >97%) used for quantification of setomimycin in the crude extract of Streptomyces sp. RA-WS2. Figure S2. Time course of fermentation of Streptomyces sp. RA-WS2 and setomimycin production for validation of optimized conditions [file 13568_2022_1497_MOESM1_ESM.docx]

A biostatistical approach for augmenting rare bianthraquinone antibiotic production by *Streptomyces* sp. RA-WS2 using Taguchi design

**Ravi Singh Manhas^1.3^, Amit Kumar^2,3^ and Asha Chaubey^*1,3^**

*^1^Fermentation & Microbial Biotechnology Division, CSIR-Indian Institute of Integrative Medicine, Canal Road, Jammu-180001 (India)*

*^2^Quality Management & Instrumentation Division, CSIR-Indian Institute of Integrative Medicine, Canal Road, Jammu-180001 (India)*

*^3^Academy of Scientific and Innovative Research, CSIR- Human Resource Development Centre, Campus Ghaziabad-201002 (India)*

**Table S1a: Medium composition for maintenance of *Streptomyces* sp. RA-WS2**

| **Ingredients** | **(g/L)** |
| --- | --- |
| Soluble starch | 10.0 |
| KNO_3_ | 2.0 |
| Casein | 0.3 |
| K_2_HPO_4_ | 2.0 |
| MgSO_4_ | 0.05 |
| NaCl | 2.0 |
| pH | 7.0±0.2 |

**Table S1b: Medium composition for pre-inoculum preparation**

| **Ingredients** | **(g/L)** |
| --- | --- |
| Soluble starch | 25.0 |
| Soyabean meal | 15.0 |
| Yeast extract | 2.0 |
| CaCO_3_ | 4.0 |
| pH | 7.0±0.2 |

**Table S1c: Medium composition for inoculum preparation**

| **Ingredients** | **(g/L)** |
| --- | --- |
| Glycerol | 10.0 |
| Calcium carbonate | 3.0 |
| K_2_HPO_4_ | 1.0 |
| Soyabean meal | 2.0 |
| MgSO_4_ | 1.0 |
| NaCl | 2.0 |
| pH | 7.0±0.2 |

**Table S1d: Components of various media used for fermentation of *Streptomyces* sp. RA-WS2 for setomimycin production**

| **S. No.** | **Medium** | **Composition in g/L** |
| --- | --- | --- |
| 1 | PM-1 | Glycerol-20.0; Soyabean meal-20; Sodium chloride-3.0 |
| 2 | PM-2 | Starch-25.0; Soyabean meal-15.0; Yeast extract-2.0; CaCO_3_ -4.0 |
| 3 | PM-3 | Dextrin-1.0; Glucose-1.0; Soyabean meal-5.0; Yeast extract-2.0; CaCO_3_ -1.50 |
| 4 | PM-4 | Glucose-50.0; Glycerol-5.0; Peptone-10.0; Malt extract-1.50; Soyabean meal-10.0; MgSO_4_.7H_2_O-1.0; CaCO_3_ -5.0; Beef extract-5.0 |
| 5 | PM-5 | Glucose-2.0; Soyabean meal-10.0; Starch-30.0; Corn Steep liquor-1.0; Peptone-3.0; CaCO_3_ -5.0 |
| 6 | PM-6 | Starch-10.0; KNO_3_-2.0; Casein-0.30; K_2_HPo_4_-2.0; MgSO_4_-0.05, NaCl-2.0 |
| 7 | PM-7 | Starch-10.0; CaCO_3_ -3.0; K_2_HPO_4_ -1.0; (NH_4_)_2_ SO_4_; -2.0 MgSO_4_-1.0; NaCl-1.0 |
| 8 | PM-8 | Starch-10.0; Casein-10.0; Peptone-1.0; Yeast extract-1.0 |
| 9 | PM-9 | KNO_3_-1.0; K_2_HPO_4_ -0.5; MgSO_4_-0.5; NaCl-0.5g; FeSO_4_-0.010; Starch-20.0 |
| 10 | PM-10 | Oatmeal-10.0; MgSO_4_-1.0; KCl-1.0; KH_2_PO_4_-0.5; K_2_HP0_4_-0.5; CaCl_2_-2.0; Yeast extract-4.0; Malt extract-4.0 |
| 11 | PM-11 | Starch-24.0; Dextrin-46.0; Yeast extract-5.0; Peptone-4.0; Soyabean meal-5.0; K_2_HPO_4_-1.0; CaCO_3_ -1.0; MgSO_4_-1.0 |
| 12 | PM-12 | yeast extract-1.0; beef extract-1.0; casamino acids-2.0; glucose- 10.0 |

**Table S2: Gradient elution of mobile phase for quantification of setomimycin by HPLC**

| **Time** | **Solution (A) %** | **Solution (B) %** |
| --- | --- | --- |
| 0 to 15min | 90 | 10 |
| 15 to 25 min | 70 | 30 |
| 25 to 35min | 40 | 60 |
| 35 to 45 min | 10 | 90 |
| 45 to 50 min | 90 | 10 |

**Table S3: Assignment of experimental conditions in the orthogonal array design L9**

**for culture in 30L fermenter**

| **Run** | **Factor 1: Glycerol (g/l)** | **Factor 2:**  **Soyabean meal (g/l)** | **Factor 3:**  **Air (LPM)** | **Factor 4: Agitation (RPM)** |
| --- | --- | --- | --- | --- |
| 1 | 150 | 5 | 10 | 200 |
| 2 | 150 | 2.5 | 20 | 150 |
| 3 | 100 | 5 | 20 | 100 |
| 4 | 150 | 7.5 | 15 | 100 |
| 5 | 50 | 5 | 15 | 150 |
| 6 | 100 | 7.5 | 10 | 150 |
| 7 | 100 | 2.5 | 15 | 200 |
| 8 | 50 | 2.5 | 10 | 100 |
| 9 | 50 | 7.5 | 20 | 200 |


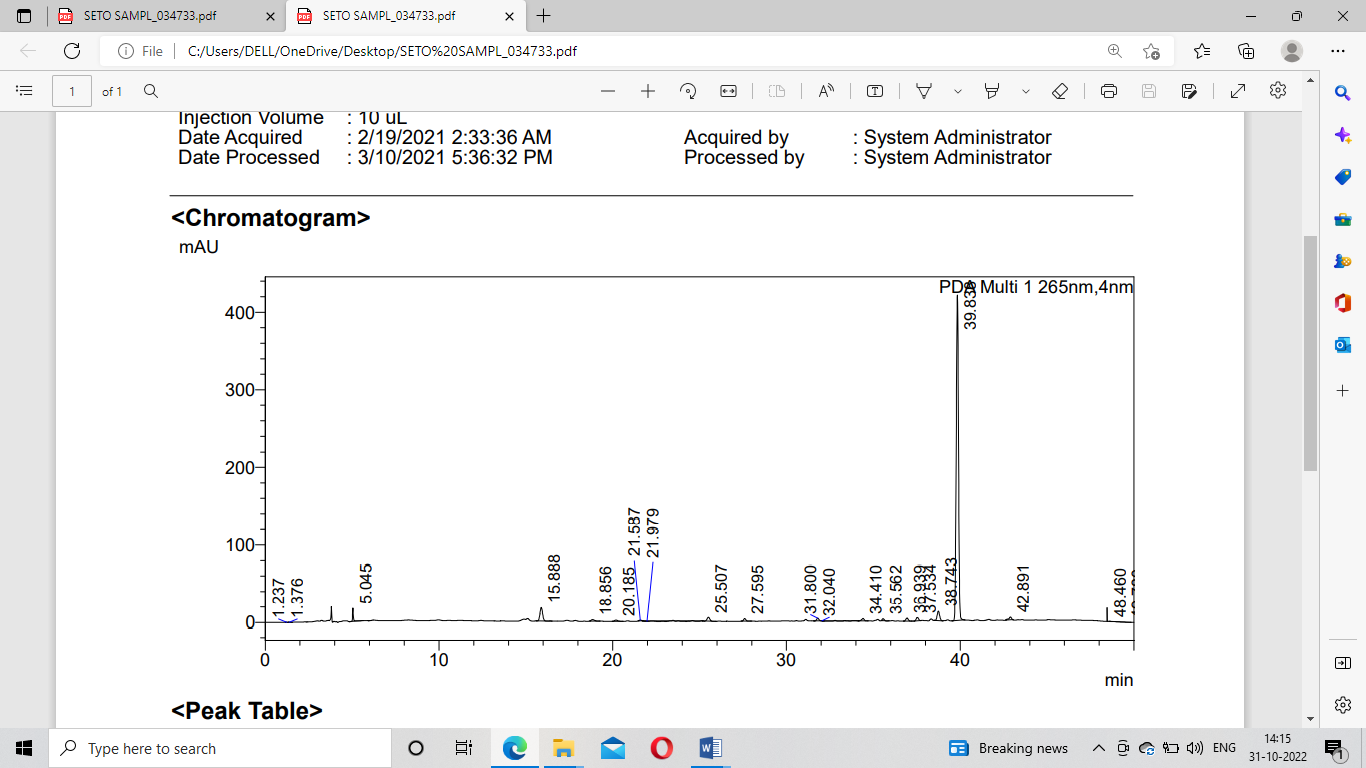


Setomimycin

**Figure S 1(a): HPLC chromatogram of crude extract of *Streptomyces* sp. RA-WS2**

**
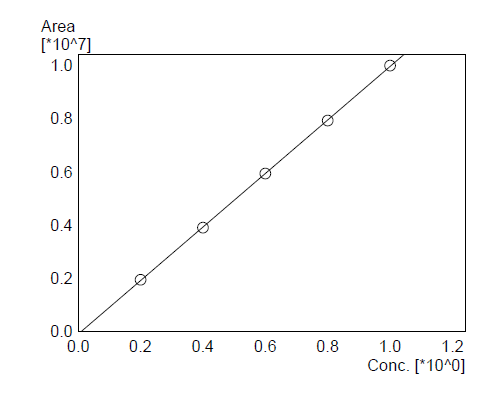
**

**R^2^=0.975**

**Figure S 1(b): Standard curve of setomimycin (purity >97%) used for quantification of setomimycin in the crude extract of *Streptomyces* sp. RA-WS2**

**Figure S2 Time course of fermentation of *Streptomyces* sp. RA-WS2 and setomimycin production for validation of optimized conditions**
